# Trends in Poliovirus Seroprevalence in Kano State, Northern Nigeria

**DOI:** 10.1093/cid/ciy637

**Published:** 2018-10-30

**Authors:** Harish Verma, Zubairu Iliyasu, Kehinde T Craig, Natalie A Molodecky, Utibeabasi Urua, Binta Wudil Jibir, Garba Dayyabu Gwarzo, Auwalu U Gajida, Sharla McDonald, William C Weldon, M Steven Oberste, Fiona Braka, Pascal Mkanda, Roland W Sutter

**Affiliations:** 1 World Health Organization, Geneva, Switzerland; 2 Department of Community Medicine, Aminu Kano Teaching Hospital & Bayero University, Kano, Nigeria; 3 World Health Organization, Abuja, Nigeria; 4 National Primary Health Care Development Agency, Abuja, Nigeria; 5 Department of Pediatrics, Murtala Mohammed Specialist Hospital, Kano, Nigeria; 6 Department of Pediatrics, Aminu Kano Teaching Hospital & Bayero University, Kano, Nigeria; 7 IHRC, Inc, Atlanta, Georgia; 8 Centers for Disease Control and Prevention, Atlanta, Georgia

**Keywords:** poliomyelitis, seroprevalence, inactivated poliovirus vaccine, Kano state, Nigeria

## Abstract

**Background:**

Kano state has been a protracted reservoir of poliovirus in Nigeria. Immunity trends have been monitored through seroprevalence surveys since 2011. The survey in 2015 was, in addition, intended to assess the impact of use of inactivated poliovirus vaccine (IPV).

**Methods:**

It was a health facility based seroprevalence survey. Eligible children aged 6-9, 12-15 and 19-22 months of age brought to the paediatrics outpatient department of Murtala Mohammad Specialist Hospital between 19 October and 6 November 2015, were screened for eligibility. Eligible children were enrolled after parental consent, history taken, physical examination conducted, and a blood sample collected to test for neutralizing antibody titres against the three poliovirus serotypes.

**Results:**

Overall, 365 results were available in the three age groups. In the 6-9-month-old age group, the seroprevalence was 73% (95% confidence interval [CI] 64-80%), 83% (95% CI 75-88%), and 66% (95% CI 57-73%) for serotypes 1, 2, and 3, respectively. In the 12-15- and 19-22-month-old age groups, seroprevalence was higher but still remained <90% across serotypes. Seroprevalence to serotypes 1 and 3 in 2015 was similar to 2014; however, for serotype 2 there was a significant improvement. IPV received in supplemental immunization activities was found to be a significant predictor of seropositivity among 6-9-month-old infants for serotypes 1 and 2.

**Conclusions:**

Seroprevalence for serotypes 1 and 3 remains low (<80%) in 6-9-month-olds. This poses a significant risk for poliovirus spread if reintroduced into the population. Efforts to strengthen immunization coverage are imperative to secure and sustain high population immunity.

Since the launch of the Global Polio Eradication Initiative (GPEI) in 1988, tremendous progress has been made toward the goal of global polio eradication. The number of polio cases has been reduced by more than 99.99%, and the number of endemic countries has been reduced from 125 to 3—Nigeria, Pakistan, and Afghanistan [1]. Moreover, wild poliovirus type 2 (WPV2) has been certified globally eradicated [2], and 5 years have passed since the last WPV3 case was detected (from Nigeria in November 2012), leaving only WPV1 in circulation.

Over the past 5 years, Nigeria has demonstrated substantial progress in reducing endemic poliovirus transmission—from 122 reported WPV cases in 2012 (across 13 northern states) to 4 reported WPV cases in 2016 (confined to Borno state). Since 2012, Nigeria has reported 185 WPV cases, of which 26% were from Kano state, which has traditionally been regarded as the epicenter of poliovirus transmission in Nigeria. In addition to achieving the interruption of WPV, Nigeria has had to deal with cocirculation of vaccine-derived poliovirus cases of serotype 2 (cVDPV2), arising due to the use of live Sabin viruses in the oral poliovirus vaccine (OPV). The number of cVDPV2 cases peaked in Nigeria in 2009, with an annual total of 155 cases. Since that surge, 105 cVDPV2 cases have been reported (as of December 2017), 33 of which were reported in Kano state [3].

The GPEI has heavily relied on the use of OPV for eradication of poliomyelitis. Only trivalent OPV (tOPV) was available for supplementary immunization activities (SIAs) until 2004, after which additional options of type-specific monovalent OPVs (mOPV1, mOPV2, mOPV3) and bivalent OPV (bOPV 1 + 3) became available in 2005 and 2009, respectively [4]. The bivalent and monovalent formulations were increasingly used in SIAs due to their higher type-specific efficacy demonstrated in developing countries [5, 6]. In routine immunization (RI), tOPV was historically used in all OPV-using countries until April 2016, when it was replaced by bOPV in a globally synchronized initiative.

Despite the progress achieved through the use of OPV, given the risk of cVDPVs, the program has started phasing out the use of OPV starting with serotype 2 (through the tOPV-bOPV switch) and will eventually stop OPV use altogether. The World Health Organization (WHO) no longer recommends only OPV for polio eradication; inactivated poliovirus vaccine (IPV) has been introduced into RI to maintain immunity against serotype 2 poliovirus and used through SIAs in endemic/outbreak settings to accelerate the interruption of WPV/cVDPV2 transmission. In Nigeria, OPV was exclusively used in SIAs until 2014. In 2014–2015, IPV was added to OPV SIAs in selected high-risk parts of Borno, Yobe, and Kano states as a 1-time opportunity. In RI, IPV was introduced nationwide as 1 full dose at the third RI contact from April 2015 onward, but coverage remained low, especially in the Northern states [7].

Kano state has been the most important reservoir of poliovirus in Nigeria, and monitoring the immunity profile in Kano has always been a priority. Serial poliovirus seroprevalence surveys have been conducted in Kano since 2011 [8, 9] to track the progress in this state. This seroprevalence survey in 2015 was conducted to monitor the immunity trends and to assess the impact of IPV use in SIAs and introduction in RI in the state.

## METHODS

The study objectives were to assess the seroprevalence for poliovirus serotypes 1, 2, and 3 in 6- to 9-, 12- to 15-, and 19- to 22-month age groups in the Kano Metropolitan Area (KMA); to assess the change in seroprevalence from the previous surveys in 2013 and 2014 in the 6-to 9-month age group; to evaluate the impact of IPV through RI and SIAs on seroprevalence; and to evaluate potential risk factors for low seroprevalence.

The study was a health facility–based design whereby parents of potentially eligible children brought to the pediatrics outpatient department (OPD) of Murtala Mohammad Specialist Hospital (MMSH) were approached for participation of their child in the study. The children fulfilling the basic eligibility criteria of age and residential area were enrolled following parental consent.

Three age groups of 6- to 9-, 12- to 15-, and 19- to 22-months were selected to align with the study objectives of comparing seroprevalence in different age groups, trends in seroprevalence over the years, and assessing the impact of an IPV dose through RI or SIA.

MMSH was selected because it is the largest hospital in the KMA in terms of turnover and has a high proportion of patients who are children. Experience from the previous surveys [8, 9] confirmed that the sample from this hospital was well distributed and highly representative of the 8 highest risk local government areas (LGAs) of interest.

The Nigerian National RI schedule recommends OPV (tOPV at the time of this study and bOPV since the switch in April 2016) doses at birth and 6, 10, and 14 weeks [10]. In addition, multiple SIAs are conducted under the polio eradication efforts across the country, being identified as SIA doses for this study. Based on the SIA calendar, the study participants in Kano were eligible to receive 3–6 tOPV, 1–2 bOPV, and 0–1 IPV doses in the 6- to 9-month age group; 7–9 tOPV, 3–6 bOPV, and 1 IPV doses in the 12- to 15-month age group; and 9 tOPV, 10–12 bOPV, and 1 IPV doses in the 19- to 22-month age group.

A total sample size of 285 children was required for this study (95 children in each of the 3 age groups) to provide sufficient precision to estimate seroprevalence to all 3 poliovirus serotypes. We based the sample size calculation on the lowest seroprevalence value of 57% (for serotype 2 in 19- to22-month-old children, obtained from the previous survey in October 2014 [8], a 95% confidence level, and a precision of ±10%. Twenty-five percent inflation was applied to account for possible exclusions. This resulted in a sample size of 120 children in each age group for a total of 360 children.

Children brought to the OPD of MMSH, fulfilling the age requirement and residing in any of the LGAs of KMA for at least 1 month, were screened further, and their parents were approached for informed consent. All who fulfilled the eligibility criteria and consented were enrolled, except those with a contraindication to venepuncture, serious acute illness/indication for hospitalization, or diagnosed or suspected congenital immunodeficiency disorder in the child or an immediate family member.

Enrollment began on 19 October 2015 and was completed on 6 November 2015. The procedures included completion of the relevant questionnaire with history of vaccine doses, measurement of weight and height/length, collection of blood sample, and other details. Regulations governing research involving humans were followed throughout the study. The Institutional Review Board of Aminu Kano Teaching Hospital and the Ethics Review Committee of WHO approved the project.

One milliliter of blood was collected through venipuncture from each participant. Blood was allowed to clot and the serum separated by centrifugation. Sera were stored at −20°C until shipped to the laboratory. After all samples were collected, sera were shipped to the Centers for Disease Control and Prevention in Atlanta, Georgia. Sera were tested in triplicate for levels of neutralizing antibody against poliovirus types 1, 2, and 3 using a modified microneutralization assay in dilutions from 1:8 to 1:1024 [11]. The antibody titers were reported on the log_2_ scale (with the lower and upper limits of quantification at 2.5 and 10.5 log_2_, respectively) and were converted to reciprocal titers for analysis. For seropositivity, reciprocal antibody titers of ≥8 were regarded as positive (ie, detectable titer).

Study participants’ dosage histories of OPV and IPV were based on parental recall, as immunization cards were not available for most children enrolled in the survey. The doses received through RI and SIA were reported separately with all OPV and IPV doses considered except the final dose if it was administered on the day of blood collection. Measurements of height and weight were used to assess nutritional status and determine malnutrition (ie, stunting and wasting) by comparing the child’s measurements to the WHO Child Growth Standards reference population based on the WHO Multicentre Growth Reference Study [12] using WHO Anthro Software [13]. Nutritional status was classified into normal, moderate, or severe malnutrition based on the Z-score (ie, the number of standard deviations [SDs] a given data point lies from the mean). The classification was defined in terms of the following Z-score (ie, SD) units below the reference mean: normal <2 SD, moderate 2.0–2.99 SD, and severe ≥3 SD.

Data collected with questionnaires were double entered into a database using CSPro software (version 5.0) [14]. The association between dichotomous potential predictors and seroprevalence was assessed using χ^2^ and Fisher exact tests, as appropriate (based on the number of observations). To assess trends in seroprevalence for ordinal variables, the Cochrane-Armitage test was used. All risk factors with *P* < .1 in the univariable analysis were considered in a multivariable model. Multivariable analysis was conducted using logistic regression to estimate adjusted odds ratios and assess the association of risk factors with seroprevalence. For each serotype, the most parsimonious yet best-fitting model was selected based on the Akaike information criterion using a stepwise addition approach [15]. *P* values < .05 were considered statistically significant. To assess the additional impact of IPV on seroprevalence, the analysis was restricted to children who received at least 3 prior OPV doses. All data analysis was conducted using R (R Foundation) version 3.2.3 (2015) [16].

## RESULTS

In the in 6- to 9-, 12- to 15-, and 19- to 22-month age groups, respectively, 128, 118, and 120 children were enrolled, resulting in 366 children enrolled overall. Serology results were available for 128, 118, and 119 children in the 6- to 9-, 12- to 15-, and 19- to 22-month age groups, respectively. Therefore, the analysis included information on 365 children in this population.

The demographic characteristics of the study population are presented in Table 1. The proportion of male and female participants was similar in the 6- to 9-month age group, but there were more males in the 12- to 15- and 19- to 22-month age groups. Mother’s education for the 12- to 15-month-olds was lower than in the other 2 age groups. Overall, 24%–30% of patients had moderate or severe wasting and 14%–30% had moderate or severe stunting.

**Table 1.** Baseline Characteristics of Study Population, Kano, Nigeria, 2015

Characteristic	Age 6–9 Months (n = 128)	Age 12–15 Months (n = 118)	Age 19–22 Months (n = 119)
	n/N (%)	n/N (%)	n/N (%)
Gender			
Female	65/128 (50.8)	46/118 (39.0)	54/119 (45.4)
Male	63/128 (49.2)	72/118 (61.0)	65/119 (54.6)
Mother’s education			
Primary or less	62/128 (48.4)	72/118 (61.0)	56/119 (47.1)
Secondary/Tertiary	66/128 (51.6)	46/118 (39.0)	63/119 (52.9)
Father’s education			
Primary or less	37/128 (28.9)	36/118 (30.5)	32/119 (26.9)
Secondary/Tertiary	91/128 (71.1)	82/118 (69.5)	82/118 (73.1)
No. of children aged <5 years			
1–2	95/127 (74.8)	92/118 (78.0)	91/119 (76.5)
>2	32/127 (25.2)	26/118 (22.0)	28/119 (23.5)
Wasting			
1, no	96/127 (75.6)	87/118 (73.7)	83/119 (69.7)
2, moderate	19/127 (15.0)	19/118 (16.1)	26/119 (21.8)
3, severe	12/127 (9.4)	12/118 (10.2)	10/119 (8.4)
Stunting			
1, no	109/127 (85.8)	96/118 (81.4)	83/119 (69.7)
2, moderate	13/127 (10.2)	11/118 (9.3)	19/119 (16.0)
3, severe	5/127 (3.9)	11/118 (9.3)	17/119 (14.3)
Routine OPV doses			
0	22/128 (17.2)	24/118 (20.3)	26/118 (22.0)
1	10/128 (7.8)	9/118 (7.6)	6/118 (5.1)
2	7/128 (5.5)	10/118 (8.5)	10/118 (8.5)
3	13/128 (10.2)	17/118 (14.4)	8/118 (6.8)
4	76/128 (59.4)	58/118 (49.1)	68/118 (57.6)
SIAs OPV doses			
0	17/127 (13.4)	9/117 (7.7)	1/118 (0.8)
1–3	40/127 (31.5)	15/117 (12.8)	13/118 (11.0)
4–6	60/127 (47.2)	25/117 (21.4)	15/118 (12.7)
≥7	10/127 (7.9)	68/117 (58.1)	89/118 (75.4)
Total doses (RI and SIAs)			
0	7/127 (5.5)	3/117 (2.6)	0/118 (0.0)
1–3	11/127 (8.7)	11/117 (9.4)	5/118 (4.2)
4–6	41/127 (32.3)	13/117 (11.1)	14/118 (11.9)
≥7	68/127 (53.5)	90/117 (76.9)	99/118 (83.9)
IPV in RI			
Yes	38/122 (31.1)	19/112 (17.0)	5/117 (4.3)
No	84/122 (68.8)	93/112 (83.0)	112/117 (95.7)
IPV in SIA			
Yes	20/128 (15.6)	53/117 (45.3)	71/118 (60.2)
No	108/128 (84.4)	64/117 (54.7)	47/118 (39.8)

Abbreviations: IPV, inactivated poliovirus vaccine; n, number of children; N, total number of children; OPV, oral poliovirus vaccine; RI, routine immunization; SIA, supplementary immunization activity.

**Figure 1.**
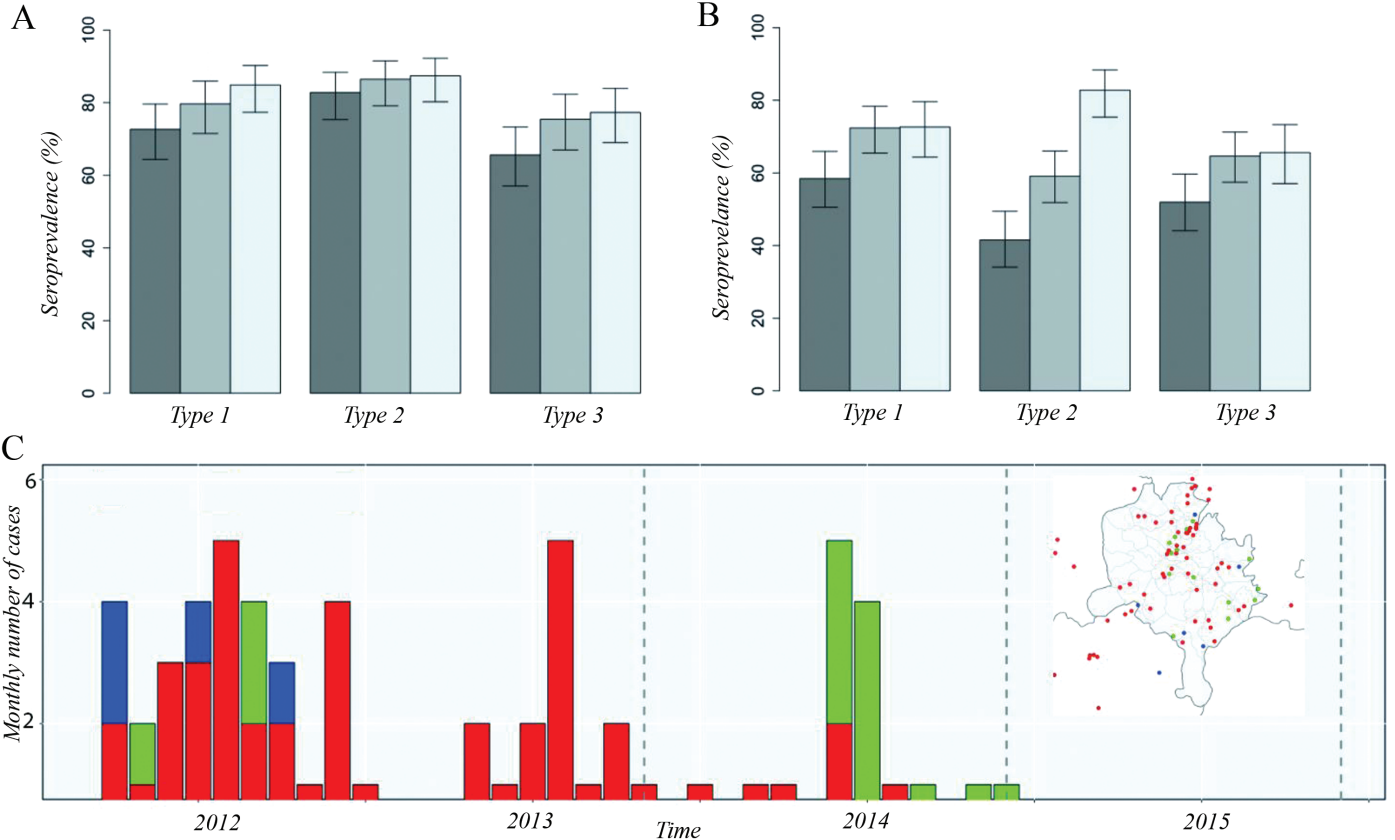
Seroprevalence and 95% confidence intervals (CIs) by poliovirus serotype, age group, and survey, Kano state, northern Nigeria, 2015. *(A*) Seroprevalence and 95% CIs by poliovirus serotype and age group, Kano state, northern Nigeria, 2015. *(B*) Seroprevalence in the 6- to 9-month age group in the 2013, 2014, and 2015 seroprevalence surveys in Kano state. *(C*) Monthly number of wild poliovirus serotype 1 (WPV1), circulating vaccine-derived serotype 2 (cVDPV2), and WPV serotype 3 (WPV3) cases between 2012 and 2014. There have been no cases in Kano state since 2015. Inset map displays the spatial distribution of cases between 2012 and 2015. Colors indicate serotype, with red corresponding to WPV1, green to cVDPV2 and blue to WPV3. 
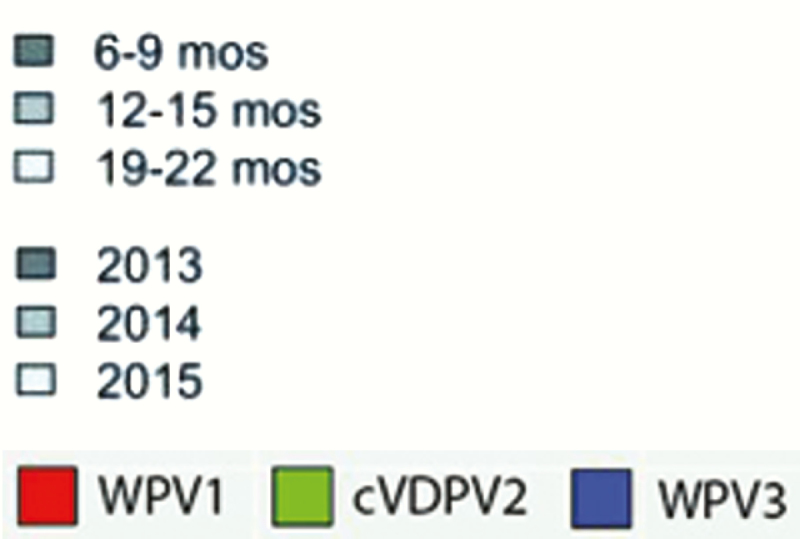

The seroprevalence in those aged 6- to 9-months was 72.7% (95% confidence interval [CI], 64.4–79.6), 82.8% (95% CI, 75.3–88.4), and 65.6% (95% CI, 57.0–73.3) for serotypes 1, 2, and 3, respectively. In 12- to 15-month-old children, the seroprevalence was higher, 79.7% (95% CI, 71.5–85.9), 86.4% (95% CI, 79.1–91.5), and 75.4% (95% CI, 66.9–82.3), for serotypes 1, 2, and 3, respectively; with the highest seroprevalence in 19- to 22-month-old children (84.9% [95% CI, 77.3–90.2], 87.4% [95% CI, 80.2–92.2], and 77.3% [95% CI, 69.0–83.9] for serotypes 1, 2, and 3, respectively; Figure 1). Overall, seroprevalence was lowest for serotype 3 and highest for serotype 2 across all age groups. There was a trend of increase in seroprevalence for all 3 poliovirus types with increasing age, particularly for serotypes 1 (*P* = .061) and 3 (*P* = .085), albeit not significant.

In the 6- to 9-month group, seroprevalence to serotypes 1 and 3 in 2015 was consistent with seroprevalence in 2014 (*P* = .957 and *P* = .858, respectively); however, for serotype 2 there was a significant increase from 59% (95% CI, 52%–66%) in 2014 to 83% (95% CI, 75%–88%; *P* < .001) in 2015. Seroprevalence to serotypes 1, 2, and 3 was significantly higher in 2015 compared to 2013 (*P* = .013, *P* < .001, and *P* = .020, respectively). Seroprevalence in patients aged 19- to 22-months in 2015 was similar to what was observed in 2014 for serotypes 1 and 3 and significantly higher for serotype 2 (*P* < .001; Figure 1).

The univariate analysis of potential predictors of seropositivity among those aged 6- to 9-months is presented in Table 2. Increased father’s educational status demonstrated a positive trend with seroprevalence; however, significance was only achieved for serotype 1. Stunting was significantly associated with low seroprevalence for type 2 and a significant negative trend was demonstrated for serotypes 1 and 3. The most consistent determinant of seropositivity was the number of OPV doses received by the child. There was a positive trend for all 3 serotypes with increasing number of RI doses and total OPV doses received by the participants. For SIA doses, the trend achieved significance only for serotypes 2 and 3. Results were consistent through the multivariate analysis (ie, after adjusting for potential confounders; Table 3).

**Table 2.** Univariate Analysis of Predictors of Seropositivity Among Patient Aged 6–9 Months by Demographic or Other Attributes, Kano State, Northern Nigeria, 2015

Characteristics	Serotype 1			Serotype 2		Serotype 3	
	N	n	% (95% CI)	n	% (95% CI)	n	% (95% CI)
Seroprevalence	128	93	72.7 (64.4–79.6)	106	82.8 (75.3–88.4)	84	65.6 (57.0–73.3)
Gender^a^							
Female	65	49	75.4 (63.7–84.2)	52	80.0 (68.7–87.9)	46	70.8 (58.8–80.4)
Male	63	44	69.8 (57.6–79.8)	54	85.7 (75.0–92.3)	38	60.3 (48.0–71.5)
Mother’s education^a^							
Primary or less	62	44	71.0 (58.7–80.8)	48	77.4 (65.6–86.0)	39	62.9 (50.5–73.8)
Secondary/Tertiary	66	49	74.2 (62.6–83.2)	58	87.9 (77.9–93.7)	45	68.2 (56.2–78.1)
Father’s education^a,b^							
Primary or less	37	20	54.0 (38.4–69.0)	27	73.0 (57.0–84.6)	22	59.5 (43.5–73.6)
Secondary/Tertiary	91	73	80.2 (70.9–87.1)	79	86.8 (78.3–92.3)	62	68.1 (58.0–76.8)
No. of children aged <5 years^a^							
1–2	95	70	73.7 (64.0–81.5)	79	83.2 (74.4–89.4)	62	65.3 (55.3–74.1)
>2	32	23	71.9 (54.6–84.4)	26	81.2 (64.7–91.1)	22	68.7 (51.4–82.0)
Wasting^c^							
1, no	96	67	69.8 (60.0–78.1)	80	83.3 (74.6–89.5)	61	63.5 (53.6–72.5)
2, moderate	19	16	84.2 (62.4–94.5)	16	84.2 (62.4–94.5)	14	73.7 (51.2–88.2)
3, severe	12	9	75.0 (46.8–91.1)	9	75.0 (46.8–91.1)	8	66.7 (39.1–86.2)
Stunting^b,c,d^							
1, no	109	83	76.1 (67.3–83.2)	91	83.5 (75.4–89.3)	77	70.6 (61.5–78.4)
2, moderate	13	7	53.8 (29.1–76.8)	10	76.9 (49.7–91.8)	4	30.8 (12.7–57.6)
3, severe	5	2	40.0 (11.8–76.9)	4	80.0 (37.5–96.4)	2	40.0 (11.8–76.9)
Routine OPV doses^b,c,d,e^							
0	22	8	36.4 (19.7–57.0)	9	40.9 (23.3–61.3)	6	27.3 (13.1–48.1)
1	10	5	50.0 (23.7–76.3)	8	80.0 (49.0–94.3)	5	50.0 (23.7–76.3)
2	7	6	85.7 (48.7–97.4)	5	71.4 (35.9–91.8)	2	28.6 (8.2–64.1)
3	13	11	84.6 (57.8–95.7)	11	84.6 (57.8–95.7)	10	76.9 (49.7–91.8)
4	76	63	82.9 (72.9–89.7)	73	96.0 (89.0–98.6)	61	80.3 (70.0–87.7)
SIAs OPV doses^c,d,e^							
0	17	9	52.9 (31.0–73.8)	8	47.1 (26.2–69.0)	6	35.3 (17.3–58.7)
1–3	40	29	72.5 (57.2–83.9)	35	87.5 (73.9–94.5)	24	60.0 (44.6–73.6)
4–6	60	44	73.3 (61.0–82.9)	52	86.7 (75.8–93.1)	44	73.3 (61.0–82.9)
≥7	10	10	100.0 (72.2–100.0)	10	100.0 (72.2–100.0)	10	100.0 (72.2–100.0)
Total doses (RI and SIAs)^b,c,d,e^							
0	7	1	14.3 (2.6–51.3)	1	14.3 (2.6–51.3)	0	0.0 (0.0–35.4)
1–3	11	3	27.3 (9.7–56.6)	6	54.5 (28.0–78.7)	2	18.2 (5.1–47.7)
4–6	41	32	78.0 (63.3–88.0)	33	80.5 (66.0–89.8)	27	65.8 (50.5–78.4)
≥7	68	56	82.3 (71.6–89.6)	65	95.6 (87.8–98.5)	55	80.9 (70.0–88.5)

Abbreviations: CI, confidence interval; OPV, oral poliovirus vaccine; RI, routine immunization; SIA, supplementary immunization activity.

^a^Pearson χ^2^or Fisher exact test.

^b^Covariate significantly associated with seropositivity to type 1 for α = 0.05.

^c^Cochrane-Armitage test for trend.

^d^Covariate significantly associated with seropositivity to type 3 for α = 0.05.

^e^Covariate significantly associated with seropositivity to type 2 for α = 0.05.

**Table 3.** Multivariate Analysis of Predictors of Seropositivity Among Patients Aged 6–9 Months by Demographic or Other Attributes Kano State, Northern Nigeria, 2015

		Type 1		Type 2		Type 3	
Covariate	Comparison	OR (95% CI)	*P* Value	OR (95% CI)	*P* Value	OR (95% CI)	*P* Value
Father’s education	Secondary/Tertiary vs primary or less	2.76 (1.07–7.15)	.035	NA	NA	NA	NA
Stunting	Moderate and severe vs no stunting	0.44 (0.13–1.47)	.173	NA	NA	0.27 (0.08–0.89)	.034
Routine immunization	Each additional dose	1.51 (1.16–1.99)	.003	2.35 (1.67–3.54)	<.001	1.71 (1.32–2.27)	<.001
SIA	1+ vs 0 SIA doses	2.46 (0.71–8.37)	.147	11.54 (2.69–59.13)	.001	4.38 (1.31–15.6)	.018

P value indicates significance based on best-fitting model for each poliovirus serotype. .

Abbreviations: CI, confidence interval; NA, not included in final model based on Akaike information criterion; OR, odds ratio; SIA, supplementary immunization activity.

The addition of IPV in RI demonstrated a positive, albeit not significant, association with seropositivity across all serotypes (Table 4). A similar trend was observed for IPV through SIA; however, the association was significant for serotype 1 (*P* = .047) and nearly significant for serotype 2 (*P* = .058). No significant association was found for serotype 3.

**Table 4.** Additional Impact of Inactivated Poliovirus Vaccine on Seropositivity in Kano State, Northern Nigeria, 2015

		Type 1			Type 2			Type 3		
Method	Inactivated Poliovirus Vaccine	n/N	% (95% CI)	*P* Value	n/N	% (95% CI)	*P* Value	n/N	% (95% CI)	*P* Value
Routine immunization	No	52/73	71.2 (60.0–80.3)	.165	61/73	83.6 (73.4–90.3)	.255	49/73	67.1 (55.7–76.8)	.270
	Yes	32/38	84.2 (69.6–92.5)		35/38	92.1 (79.2–97.3)		30/38	78.9 (63.6–88.9)	
Supplementary immunization activity	No	81/103	78.6 (69.8–85.4)	.047	87/103	84.5 (76.2–90.2)	.058	78/103	75.7 (66.6–83.0)	.332
	Yes	108/122	88.5 (81.6–93.0)		113/122	92.6 (86.6–96.1)		99/122	81.1 (73.3–87.1)	

Numbers are for inactivated poliovirus vaccine (IPV) in routine immunization (RI) in those aged 6–9 months and IPV in supplementary immunization activity in those aged 12–15 months and 19–22 months. Analysis restricted to children who received at least 3 oral poliovirus vaccine doses.

Abbreviation: CI, confidence interval.

## DISCUSSION

The seroprevalence survey in Kano, Nigeria, provides critical information on the immunity profile against poliomyelitis in one of the highest-risk states in Nigeria. In the 6- to 9-month-old cohort, the seroprevalence remains low (<80%) for serotypes 1 and 3, creating a significant immunity gap that poses a risk for poliovirus spread if it is reintroduced into the population. Only serotype 2 seroprevalence has demonstrated improvements in 2015 over 2014, primarily due to the increased number of SIAs with serotype 2 containing tOPV (prior to OPV2 withdrawal) and the introduction of IPV in the RI schedule. Seroprevalence in the 2 older age groups was higher for all serotypes, which can be attributed to the larger number of vaccine doses with increasing age and possibility of more indirect exposure to the vaccine viruses from the environment.

History of OPV doses remains the most consistent determinant of seropositivity. An increasing number of RI OPV doses were significantly associated with increased seroprevalence for all 3 serotypes. Given that 30% of infants in the 6- to 9-month age group received <3 RI OPV doses (despite being eligible for 4 OPV doses), there is room for substantial improvement in seroprevalence through strengthening of RI. These results reemphasize the value of high vaccine coverage and strong immunization systems as components of the strategy of polio eradication and sustaining polio-free status.

There was a significantly increasing trend in seroprevalence for poliovirus serotypes 2 and 3 with increasing OPV doses through SIAs. Seroprevalence for serotype 1 demonstrated improvement with increasing number of doses; however, the trend was not significant. Despite multiple SIA campaigns being conducted in the catchment area of the seroprevalence study, 13% and 32% of children reported 0 or 1–3 SIA OPV doses, respectively. These analyses highlight persistent gaps in SIA quality.

Unsurprisingly, given the strong relationship between OPV doses through RI and SIA, the correlation between total OPV doses (routine and SIAs combined) and seroprevalence was found to be highly significant for all 3 poliovirus types. The data demonstrate that the strategy of multiple campaigns is important to sustain or improve type-specific immunity in the young cohorts. Suboptimum coverage continues to be the root cause for low seroprevalence and the impending risk of sustained poliovirus transmission. Despite a strong emphasis on improving RI coverage and achieving 100% SIA coverage in children aged <5 years, these data show 14% of 6- to 9-month-old infants did not receive any RI or SIA OPV dose. These results call for a deep introspection into the operational plan of immunization in Kano, particularly for SIAs.

Since most infants are protected due to maternal antibodies until at least 6 months of age [17], the seronegative cohort beyond 6 months constitutes a potentially susceptible pool for poliovirus infection and transmission. With the program providing multiple opportunities for vaccination both through RI and SIAs, it should be possible to achieve higher seroprevalence. Although the exact levels required for herd immunity are difficult to define in this context, seroprevalence of 73% and 66% for serotypes 1 and 3, respectively, are far below the estimated threshold levels required [18] or previously achieved in other high-risk areas [19, 20].

In addition, the study was intended to assess the impact of IPV received through RI or SIAs. Administering IPV to OPV-primed children is known to boost immunity levels [21, 22], with the potential to close immunity gaps [23]. This is supported by the results from this survey, as demonstrated by the substantial gain in seroprevalence following an IPV dose through SIA in children with previous OPV exposure. However, the full impact of IPV on seroprevalence could not be demonstrated in this population, because only 53% of children had received IPV through SIA. Again, in our sample, the addition of IPV into the RI schedule had a positive impact on seroprevalence. However, as only 31% of infants aged 6–9 months received IPV through RI and the sample size was small, it limited the inference of significance. Therefore, despite the positive impact of IPV on seroprevalence, the added benefit of IPV on immunity is highly dependent on the coverage achieved.

There were some limitations in this study. First, this was a health facility–based study; therefore, the results may not be generalizable to the entire population. The health facility–based design was adopted due to operational convenience and the security challenges associated with community-based operations in northern Nigeria. Second, the demographic and dose history variables were based on parent recall and therefore may be subject to recall error. Third, the analysis to assess the impact of IPV was restricted to children with ≥3 OPV doses to ensure the IPV and non-IPV groups were comparable. It was not possible to repeat the analysis for children with <3 OPV doses due to very low numbers with IPV in this group.

In this study, OPV and IPV were both demonstrated to have a substantial impact on seroprevalence in Kano state. Given the importance of vaccination on seroprevalence, the polio program must ensure all children are immunized. With WPV1 cases reported in Borno and cVDPV2 detected in the environment in Borno and Sokoto states as late as 2016–2017 [1, 24], Kano remains at risk of importation and transmission. Kano has been the most persistent reservoir of poliovirus transmission in Nigeria; therefore, it is essential to ensure and maintain high levels of population immunity. Any complacency at this stage poses substantial risk to the ultimate goal of global polio eradication.

## Notes

***Acknowledgments.*** We acknowledge the contributions of the entire research team involved at the study sites for different study procedures and responsibilities. We thank the administrative and management teams of the collaborating institutions: Aminu Kano Teaching Hospital Kano, Murtala Mohammad Specialist Hospital Kano, the National and State Primary Health Care Development Agencies, and different levels in WHO. We express special appreciation for Deborah Moore, Yiting Zhang, William Hendley, Larin McDuffie, Mario Nicolas, and Patricia Mitchell who performed the serological assays. Finally, compliments to Rotary International for funding this project through WHO.

***Financial support.*** Support was received from WHO (using a grant from Rotary International, IPPC PP14BMF0003).

***Supplement sponsorship.*** This article appears as part of the supplement “Polio endgame and beyond: Vaccine choices, transmission dynamics, and surveillance implications,” sponsored by The Bill & Melinda Gates Foundation.

***Potential conflicts of interest.*** All authors: No reported conflicts of interest. All authors have submitted the ICMJE Form for Disclosure of Potential Conflicts of Interest. Conflicts that the editors consider relevant to the content of the manuscript have been disclosed.
